# Advancing women’s mental health services in Qatar

**DOI:** 10.1192/bji.2025.10070

**Published:** 2026-05

**Authors:** Majid Alabdulla, Javed Latoo, Yasser Saeed Khan, Yousaf Iqbal, Sazgar Abdullah Hamad, Ovais Wadoo

**Affiliations:** 1 Chairman, Department of Psychiatry, Hamad Medical Corporation, Doha, Qatar; 2 Senior Consultant, Department of Psychiatry, Hamad Medical Corporation, Doha, Qatar; 3 Senior Consultant, Department of Psychiatry, Hamad Medical Corporation, Doha, Qatar; 4 Consultant, Department of Psychiatry, Hamad Medical Corporation, Doha, Qatar; 5 Consultant, Department of Psychiatry, Hamad Medical Corporation, Doha, Qatar; 6 Clinical Professor, Department of Psychiatry, College of Medicine, Qatar University, Doha, Qatar.

**Keywords:** Women’s health, mental health services, mental health policy, gender-sensitive care, perinatal psychiatry

## Abstract

This article provides a narrative overview of the development of women’s mental health services in Qatar. The country has made notable advancements, driven by progressive health policies and a focus on gender-sensitive care. Key initiatives include the development of specialised services, the integration of mental health into primary care and the implementation of targeted training programmes for healthcare professionals. The establishment of a fellowship programme in women’s mental health and the incorporation of gender considerations into national clinical practice guidelines further underscore the country’s commitment. Addressing remaining gaps through innovation, inclusivity and collaboration will be vital to ensuring comprehensive mental healthcare for all women.

Women’s mental health is shaped by a complex interplay of biological, psychological and sociocultural factors. Women are at increased risk of developing common mental disorders, particularly depression and anxiety, with over 20% affected during the perinatal period,^1^ and a higher lifetime risk compared with men. Hormonal fluctuations, gender-based violence, reproductive health challenges and caregiving responsibilities all contribute to this vulnerability. These issues are especially pertinent in the Arab world, where cultural expectations and social norms significantly influence women’s mental health experiences. Gender roles, stigma surrounding mental illness, and the scarcity of specialised services often pose barriers to help-seeking and access to appropriate care. In this context, adopting a gender-sensitive approach that acknowledges and addresses the unique mental health needs of women is not merely advantageous but essential.

## Historical and regional context

To understand Qatar’s progress in this area, it is important to consider the broader historical and regional context. Historically, mental health service provision across much of the Eastern Mediterranean region has been constrained by limited access, underdeveloped community services and a lack of trained professionals. In 2005, only 63.6% of countries in the region offered treatment for severe mental disorders at the primary care level, and the average number of psychiatrists per 100 000 population stood at just 0.95.^2^ Moreover, disorders disproportionately affecting women, such as depression and somatisation, were frequently under-recognised in general medical settings. Often, women present with physical complaints that mask underlying psychological distress, a pattern influenced by stigma and limited awareness of mental illness. This is partly shaped by cultural norms that make it difficult, particularly for women, to openly express emotional difficulties. In Arab societies, women face significant cultural barriers to seeking psychological help, including fear of stigma, threats to marital prospects and repercussions within their family.^3^

Against this backdrop, Qatar has taken a markedly different path, investing significantly in the development of a comprehensive, integrated and gender-sensitive mental health system. Key advancements include the expansion of women-focused services in both primary and secondary care settings, the integration of perinatal mental health into national clinical guidelines, the establishment of virtual and community outreach services and the introduction of a national fellowship programme in women’s mental health.

This article provides a narrative overview of the development of women’s mental health services in Qatar, reflecting the country’s emerging role as a regional leader and its ongoing commitment to improving access, quality and cultural relevance of care for women. The policy and legislative foundations enabling these advancements, such as the Qatar National Mental Health Strategy and mental health legislation, are also reviewed.^4,^ ^5^

## Qatar: demographics and healthcare structure

The State of Qatar is a small peninsular nation located in the Arabian Gulf. As a high-income country with a population of approximately 2.7 million, Qatar is among the wealthiest nations globally in terms of per capita gross domestic product (GDP). Arabic is the official language and Islam is the state religion. Foreign nationals constitute over 80% of the population, the majority of whom are men.^6^ This gender imbalance is largely driven by the influx of male migrant workers employed in the construction and industrial sectors, resulting in an overall male:female ratio of approximately 3:1. However, among Qatari citizens and long-term residents, the gender distribution is more balanced, underscoring the importance of addressing women’s mental health needs across both groups.

Qatar’s healthcare system is predominantly publicly funded. The Hamad Medical Corporation (HMC) serves as the principal provider of secondary and tertiary care, while the Primary Health Care Corporation (PHCC) oversees primary care services. The Qatari government has consistently prioritised healthcare investment, with mental health increasingly integrated into national health strategies.^7^

## Healthcare policy and legislation

The Qatar National Vision provides the overarching framework for the country’s development strategies, including health policy. In alignment with this vision, the State of Qatar has established policies and legislation specifically focused on mental health, drawing on international guidance to inform the development of its services and legal frameworks. A significant milestone was the launch of the Qatar National Mental Health Strategy (2013–2018),^4^ which emphasised the creation of accessible, community-based and integrated mental health services. This strategy recognised the diverse needs of different population groups, including women, and called for targeted, population-specific interventions. Building on this foundation, the National Health Strategy 2018–2022 identified mental health and well-being as one of its seven priority areas.^5^ It highlighted the importance of addressing the social determinants of mental health and expanding culturally sensitive and gender-appropriate services. Together, these strategies have played a critical role in supporting the expansion of women’s mental health services in Qatar. In 2016, Qatar enacted its first dedicated mental health legislation through a royal decree, guided by the World Health Organization’s Assessment Instrument for Mental Health Systems. However, full implementation of this legislation remains pending.^8,9^

## Overview of mental health services for women in Qatar

### Primary care services

Mental health support is increasingly accessible at the primary care level through PHCC’s health centres, which cater to both men and women. Family physicians are trained to recognise and manage common mental disorders, including depression, anxiety and somatisation, with established referral pathways to specialised mental health services when indicated. Additionally, first-line psychological interventions, such as cognitive–behavioural therapy and brief psychotherapies, are being progressively integrated into primary care. These interventions are often delivered by clinical psychologists embedded within the health centres, enhancing the availability of evidence-based mental healthcare at the community level.^10^

There are currently no publicly accessible data outlining how mental healthcare is divided between primary and secondary services in Qatar, either for the population as a whole or specifically for women. This lack of detail makes it difficult to draw direct comparisons or assess system-level trends. The National Health Strategy (2018–2022) set an explicit aim to deliver 20% of care in primary and community settings.^5^ Although exact data on progress toward this target have not been released, available reports point to a notable expansion in out-patient and community services over that period. While the overall care model in Qatar continues to develop, these efforts reflect a clear policy direction towards more integrated and accessible mental health services.

### Secondary care services

Hamad Medical Corporation is the principal provider of secondary and tertiary mental health services in Qatar. Women have access to gender-specific psychiatric care that prioritises privacy, safety and cultural sensitivity. HMC offers out-patient mental health services tailored to women’s needs, including specialised clinics focused on perinatal mental health.^11^

### Crisis services and virtual helplines

Crisis support is available to all residents in Qatar through national helpline services. In addition, a dedicated virtual women’s helpline has been established to offer confidential, culturally sensitive psychological support specifically tailored to the unique mental health needs of women.^12^

### Community mental health services

For women who are unable to access ambulatory care owing to mobility limitations, stigma or caregiving responsibilities, community mental health teams provide home-based services. These services include psychiatric assessments, medication management and psychosocial support, all delivered by multidisciplinary teams. This community-based approach promotes continuity of care, builds therapeutic trust and reduces barriers to accessing mental health support.^7^

### In-patient services

Women are provided with gender-specific in-patient services at HMC. These facilities offer trauma-informed, therapeutic environments with appropriate security and privacy considerations. In-patient care is complemented by robust discharge planning and follow-up support to ensure continuity.

### Consultation-liaison services

Consultation-liaison psychiatry services have been integrated into various HMC hospitals, particularly in obstetrics and gynaecology departments. These teams provide timely psychiatric evaluations and interventions for women experiencing antenatal and postnatal depression, perinatal psychosis or mental health complications following miscarriage, infertility treatments or other reproductive challenges.

## Training and workforce development

In terms of workforce capacity, the mental health workforce has grown substantially in recent years. Between 2014 and 2020, the number of mental health professionals per 100 000 population almost doubled, from 13.45 to 25.28. Despite this growth, workforce numbers across all categories remain below international benchmarks for high-income countries.

Mental health training programmes for general practitioners, non-physician clinicians and other healthcare personnel working in primary care have been introduced as part of in-service skill enhancement initiatives.^13^ The mental health training delivered in Qatar was influenced by and aligned with the principles of the World Health Organization (WHO) Mental Health Gap Action Program (mhGAP), although it was not a direct replication of the mhGAP curriculum. This training provided by Maudsley International typically integrates mhGAP with other evidence-based approaches and clinical best practices from the UK and elsewhere, tailored to the needs of the local healthcare system. In addition, specialists from HMC have played a key role in sustaining mental health education within the system. They have launched a continuing professional development programme, which includes regular workshops, clinical presentations and case-based discussions.

Recognising the critical need for a skilled workforce, Qatar has launched targeted training programmes to build capacity in women’s mental health. These programmes are designed to equip mental health professionals, including psychiatrists, psychologists and nurses, with the knowledge and clinical skills needed to address the complex and often under-recognised mental health needs of women across the life course. Training in perinatal psychiatry includes modules on the identification and management of common conditions such as postpartum depression, anxiety disorders and maternal psychosis, as well as the safe use of psychotropic medication during pregnancy and breastfeeding. It also emphasises early detection, collaborative care models involving obstetrics and paediatrics, and culturally sensitive approaches to patient engagement. In parallel, trauma-informed care training focuses on building clinicians’ ability to recognise and respond to the psychological impact of trauma, including gender-based violence, migration-related stressors and early adverse experiences. This involves not only clinical assessment and intervention skills, but also fostering a service environment that prioritises safety, trust, empowerment and respect. To further support subspecialisation, a dedicated fellowship programme in women’s mental health has been established. This programme is designed to equip clinicians with the expertise required to manage complex cases, coordinate multidisciplinary care and advocate for policy reform. In doing so, it contributes to both service development and academic advancement in the field.

National clinical practice guidelines (CPGs) in Qatar have increasingly integrated gender-specific considerations. For example, CPGs on perinatal mental health outline evidence-based protocols for screening, diagnosis and treatment throughout the antenatal and postnatal periods. These guidelines align with international best practices while also incorporating culturally relevant elements, such as appropriate involvement of family members and safeguarding patient confidentiality.

## Future directions

A crucial area for development in women’s mental health services in Qatar is the establishment of dedicated mother and baby units (MBUs). MBUs are considered the gold standard for in-patient psychiatric care for mothers and their infants, providing specialised support that addresses the unique needs of both. The postpartum period is critical, as mothers are particularly vulnerable to mental health problems such as anxiety and depression, which often remain undetected and untreated.^14^ Moreover, maternal mental health problems can have profound impact on infants’ and children’s development. There is a strong association between maternal mental health diagnoses and infant mental health problems, with over half of infants admitted to MBUs showing developmental concerns or mental health symptoms.^15^ Nearly 30% of infants co-admitted to MBUs or psychiatric wards are at risk of adversity in early childhood, particularly when the mother has multiple admissions. These findings stress the importance of specialised maternal mental health services to support both mothers and their infants and improve long-term outcomes. Finally, ongoing research is needed to further consolidate evidence-based models of care tailored to the diverse needs of women in Qatar, including interventions sensitive to cultural diversity and stigma reduction. Developing comprehensive mother and baby services should be accompanied by training clinicians in gender-sensitive care and expanding community-based education to improve accessibility among women.

## Conclusion

Qatar has made significant progress in advancing women’s mental health services, driven by forward-looking health policies and a sustained commitment to gender-sensitive care. As the nation continues to develop its healthcare system, it is well-positioned to lead the region in establishing comprehensive, culturally responsive mental health services for women. Addressing remaining gaps through innovation, inclusivity and cross-sector collaboration will be essential to ensuring that all women in Qatar have equitable access to the mental health support they need and deserve.

## Data Availability

Data availability is not applicable to this article as no new data were created or analysed in this study.
